# COPAL: An ensemble of protein-ligand co-folding models enriches preferred ligand predictions for the LuxR-family of quorum sensing receptors

**DOI:** 10.64898/2026.09.04.749498

**Published:** 2026-09-09

**Authors:** Davi Nakajima An, Amy L. Schaefer, Szu-Min Chang, Bianxia Bai, Charlotte I. Mulligan, Yasuhiro Oda, E. Peter Greenberg, Frank DiMaio

## Abstract

Acyl-homoserine lactone (AHL) quorum sensing enables many species of proteobacteria to coordinate collective behaviors. In such systems, a synthase produces an AHL signal, which is sensed by a LuxR-family receptor. Despite extensive genomic annotation of LuxR homologs, preferred AHLs for most receptors remain unknown, limiting functional understanding of quorum sensing across diverse bacteria. Here, we present the COPAL (combining ordered predictions of audited ligands) pipeline, which integrates multiple protein-ligand co-folding models to identify preferred AHLs for a specific LuxR. Benchmarking on a leakage-controlled subset of 96 experimentally characterized LuxR-AHL pairs shows that COPAL places the preferred AHL within the top-6 candidates (out of 58) for 68% of receptors, outperforming every individual co-folding model. Further, inter-model agreement correlates with ranking accuracy, offering an indication of confidence. We show that COPAL resolves the specificity shift induced by three-point mutations in LasR and correctly nominates C8-HSL as the preferred ligand for the previously uncharacterized *Mesorhizobium* sp. NJ3 receptor, which we verified experimentally. Finally, we release the Ranked AHL-LuxR Prediction Hub (RALPH), comprising precomputed rankings for about 10,000 unique LuxR homologs. More broadly, COPAL shows that unweighted rank aggregation of complementary co-folding models offers a general strategy for predicting receptor-ligand specificity in data-scarce biological systems.

## Full Text Availability

The license terms selected by the author(s) for this preprint version do not permit archiving in PMC. The full text is available from the preprint server.

